# Karyotype analysis of four jewel-beetle species (Coleoptera, Buprestidae) detected by standard staining, C-banding, AgNOR-banding and CMA3/DAPI staining

**DOI:** 10.3897/CompCytogen.v6i2.2950

**Published:** 2012-04-27

**Authors:** Gayane Karagyan, Dorota Lachowska, Mark Kalashian

**Affiliations:** 1 Institute of Zoology of Scientific Center of Zoology and Hydroecology, National Academy of Sciences of Armenia, P. Sevak 7, Yerevan 0014, Armenia; 2 Department of Entomology, Institute of Zoology Jagiellonian University, Ingardena 6, 30-060 Krakow, Poland

**Keywords:** Coleoptera, Buprestidae, karyotypes, Ag-banding, C-banding, CMA_3_-staining, DAPI-staining

## Abstract

The male karyotypes of *Acmaeodera pilosellae persica* Mannerheim, 1837 with 2n=20 (18+neoXY), *Sphenoptera scovitzii* Faldermann, 1835 (2n=38–46), *Dicerca aenea validiuscula* Semenov, 1895 – 2n=20 (18+Xy_p_) and *Sphaerobothris aghababiani* Volkovitsh et Kalashian, 1998 – 2n=16 (14+Xy_p_) were studied using conventional staining and different chromosome banding techniques: C-banding, AgNOR-banding, as well as fluorochrome Chromomycin A_3_ (CMA_3_) and DAPI. It is shown that C-positive segments are weakly visible in all four species which indicates a small amount of constitutive heterochromatin (CH). There were no signals after DAPI staining and some positive signals were discovered using CMA_3_ staining demonstrating absence of AT-rich DNA and presence of GC-rich clusters of CH. Nucleolus organizing regions (NORs) were revealed using Ag-NOR technique; argentophilic material mostly coincides with positive signals obtained using CMA_3_ staining.

## Introduction

The family Buprestidae (Coleoptera) is one of the largest groups of Polyphagan beetles containing over than 14500 nominal species worldwide (Bellamy 2008a-d, 2009).Until now, karyotypes have been published for 88 species (34 from Armenia) of jewel-beetles belonging to 22 genera and 14 tribes of the subfamilies Julodinae, Polycestinae, Chrysochroinae, Buprestinae and Agrilinae (Smith and Virkki 1978; Karagyan and Kuznetsova 2000; Karagyan 2001; Karagyan et al. 2004; Karagyan and Lachowska 2007; Moura et al. 2008). In the works listed the data were obtained by study of gonads of imagos. Only one work was based on the study of hemocytes of the larva of *Chalcophora mariana* Linnaeus, 1758 (Chrysochroinae, Chrysochroini) with diploid chromosome number 2n=22 (Baragaño 1978; this work was omitted in all previous reviews). The diploid chromosome numbers (2n) in the family Buprestidae range between 12 and 46. The modal number is 2n=20 found in 16 species, 8 genera, 6 tribes and 4 subfamilies. The most frequent sex chromosome system is XX/XY. The XY system of males is diverse (Xy_p_, Xy_r_, “XY”, neo-XY and multiple X- and Y- sex chromosomes). The Xy_p_ “parachute” type is the most common and occurs in 64 species, 15 genera, 10 tribes and 4 subfamilies.

Up till now, cytogenetic studies on jewel-beetles have been carried out using conventional staining techniques mainly, with few exceptions. Karagyan (2001) reported the first data on AgNOR- staining in the karyotypes of two speciesof the genus *Sphenoptera* Dejean, 1833 and four species of *Acmaeoderella* Cobos, 1955, andMoura et al. (2008) studied karyotype of *Euchroma gigantea* Linnaeus, 1758 using Ag-banding, C-banding and fluorescent *in situ* hybridization (FISH) with a rDNA probe.

The present paper is dedicated to study of karyotypes of *Acmaeodera pilosellae persica* Mannerheim, 1837, *Sphenoptera scovitzii* Faldermann, 1835, *Dicerca aenea validiuscula* Semenov, 1895 and *Sphaerobothris aghababiani* Volkovitsh & Kalashian, 1998 using conventional and differential staining techniques. AgNO_3_-banding was used to reveal the nucleolus organizing chromosomes and to locate the NORs in them. C-banding method was used to study the distribution of constitutive heterochromatin (CH). To characterize the molecular composition of the CH chromosomes were stained with DNA-specific fluorochromes DAPI and CMA_3_ which selectively stain AT-rich and GC-rich DNA fragments, respectively. In the present study the fluorochromes CMA_3_ and DAPI were applied for the first time to jewel-beetles chromosomes.

## Material and methods

The buprestid beetles were collected in 2006 in Southern Armenia (Vayotsdzor and Syunik provinces). Males’ gonads were dissected in several drops of hypotonic 0.9% sodium citrate solution containing 0.005% colchicine and incubated for 30-45 min at room temperature. Then the gonads were fixed in 3:1 ethanol-acetic acid mixture. Karyological slides were made according to the method proposed by Rożek (1994) with minor modifications (Rożek and Lachowska 2001). Slides were examined with phase contrast optics and the best of them with well spread chromosomes were chosen for further treatments. Depending on the specific task, various staining techniques were used. First, to determine the number and morphology of chromosomes, karyotypes were examined using 4% Giemsa solution in phosphate buffer (pH 6.8). Then a series of chromosome banding techniques was applied as follows:

### The Ag-banding method (Howell and Black 1980)

The method is based on selective silver staining of NORs containing clusters of functional rRNA genes. The slides were subjected to hydrolysis in 2N formic acid for 10 min, rinsed in running water and dried. Then the slides were treated by putting of 4-5 drops of 50% aqueous silver nitrate (AgNO_3_) solution and 2 drops of colloidal developer solution (0.2 g gelatin, 10 ml distilled water and 0.1 ml concentrated formic acid – HCOOH). The slides were covered with a coverslip and incubated on a hotplate for 3-4 min at 60^0^C in a moist chamber (warmed beforehand). After rinsing in distilled water, the slides were dried and stained by 4% Giemsa solution in phosphate buffer (pH 6.8) for 10-20 sec.

### The C-banding method (Rożek 2000)

The slides were treated for 1-3 min. in 0.2N HCL at room temperature then rinsed in distilled water. Thereafter the slides were placed in 5% Ba(OH)_2_ solution at 20^0^C for approximately 3 min, then carefully rinsed in streaming and distilled water, and for 1 min in 2xSSC solution (0.3 M sodium chloride containing 0.03 M tri-sodium citrate). Then the slides were incubated in 2xSSC solution at 50^0^C for 1 hr and rinsed in distilled water. Some of these slides were stained with 4% Giemsa solution in phosphate buffer (pH 6.8) and the others were dyed with DNA-specific fluorochromes DAPI and CMA_3_.

To characterize the molecular composition of the constitutive heterochromatin, we stained the chromosomes with DNA-specific fluorochromes **CMA_3_** (the antibiotic chromomycin A_3_) and **DAPI** (4’-6-diamidino-2-phenylindol) that selectively stain GC-rich and AT-rich DNA fragments, respectively (Schweizer 1976; Donlon and Magenis 1983). Staining with fluorochromes was performed after C-banding procedure as described above. After 2xSSC treatment slides were subsequently rinsed in distilled water and dried. Then slides were immersed in McIlvaine buffer (pH 7.0) for 5 min. After this, slides were stained with CMA_3_ at a final concentration of 5 µg/ml in 10 mM McIlvaine buffer at pH 7.0 (25 µl of 96% methanol was added to 500 µl of final staining solution) for 30 min, rinsed in buffer and stained with DAPI at a final concentration of 0.4 µg/ml in 10 mM McIlvaine buffer (pH 7.0) for 5 min. After staining, slides were rinsed in distilled water and mounted in anti-fade medium consisting of 1% n-propylgallate in a 10 M McIlvaine buffer (pH 7.0) solution with 70% glycerol. To improve the fluorochrome staining, 0.5% methanol was added to the fluorescent dye (Kuznetsova et al. 2001).

The slides were analyzed and photographed with a Nikon Eclipse 400 light microscope and CCD DS-U1 camera using the software Lucia Image 5.0.

## Data resources

The data underpinning the analyses reported in this paper are deposited in the Dryad Data Repository at doi: 10.5061/dryad.hc52qb12.

## Results

### Subfamily Polycestinae. Tribe Acmaeoderini

***Acmaeodera pilosellae persica***, 2n=20, n ♂ = 9 + neo-XY.

**Figure 1A–D.**
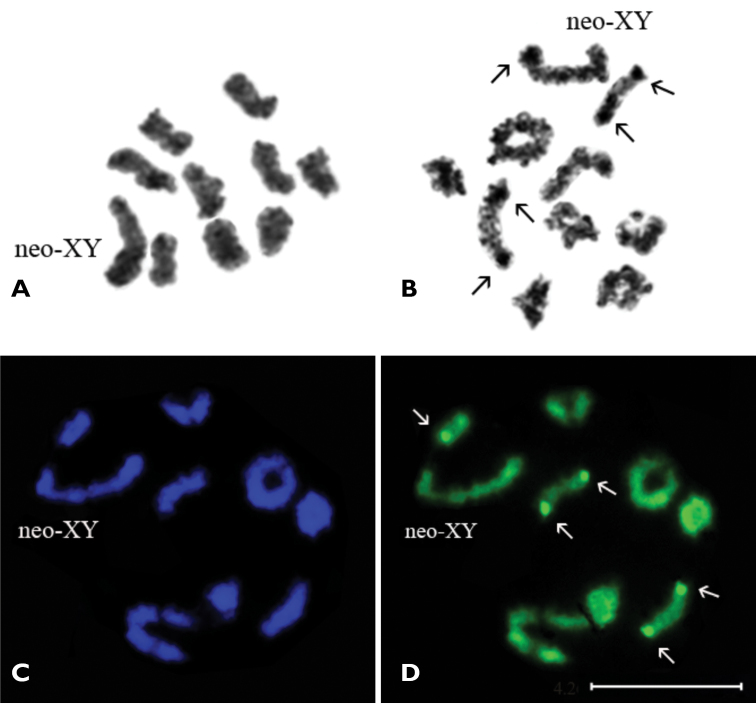
*Acmaeodera pilosellae persica*, n=9AA+ neo-XY. **A** prometaphase I/metaphase I **B** late diakinesis (Ag-banding) **C** prometaphase I (DAPI staining) **D** (CMA_3_ staining). Bar = 10 µm.

In prometaphase I/metaphase I nine autosomal bivalents formed a series of gradually decreasing sizes and large heteromorphic neo-XY sex-bivalent were observed (Fig. 1A). In late diakinesis / prometaphase I the 5–6 rod-shaped bivalents have most likely a terminal chiasma and 3–4 ring-shaped bivalents – two chiasmata (Fig. 1B). The X-chromosome seems to be submetacentric, Y-chromosome is most probably acrocentric and similar in size to the shorter arm of X-chromosome.

The C-banding technique revealed a very small amount of constitutive heterochromatin in the majority of the chromosomes (most probably, in pericentromeric regions) which do not form any distinct blocks. The DAPI staining did not reveal any positive signal and therefore absence of AT-rich clusters of DNA was demonstrated (Fig. 1C). Application of CMA_3_ staining revealed small positive signals on telomeric regions of both homologues of two or sometimes three rod-shaped autosomal bivalents. Besides, there is small ring-shaped bivalent which is brightly visible in the majority of photographs (Fig. 1D). These signals correspond to argentophilic material revealed by the AgNOR-banding technique (Fig. 1B) and probably associated with NORs. Besides, the Y-chromosome of neo-XY bivalent is dyed argentophilic.

### Subfamily Chrysochroinae. Tribe Sphenopterini

***Sphenoptera scovitzii***, 2n=38–46.

The karyotype of *Sphenoptera scovitzii* studied using Ag-banding was published earlier (Karagyan 2001) and the data on karyotype of this species obtained in that study were confirmed. Unfortunately, until now, the male diploid karyotype of the species can not be determined with certainty. Thus, it seems that karyotype consists of 38–46 chromosomes, most probably of 46; the sex chromosomes could not be identified.

**Figure 2A–F.**
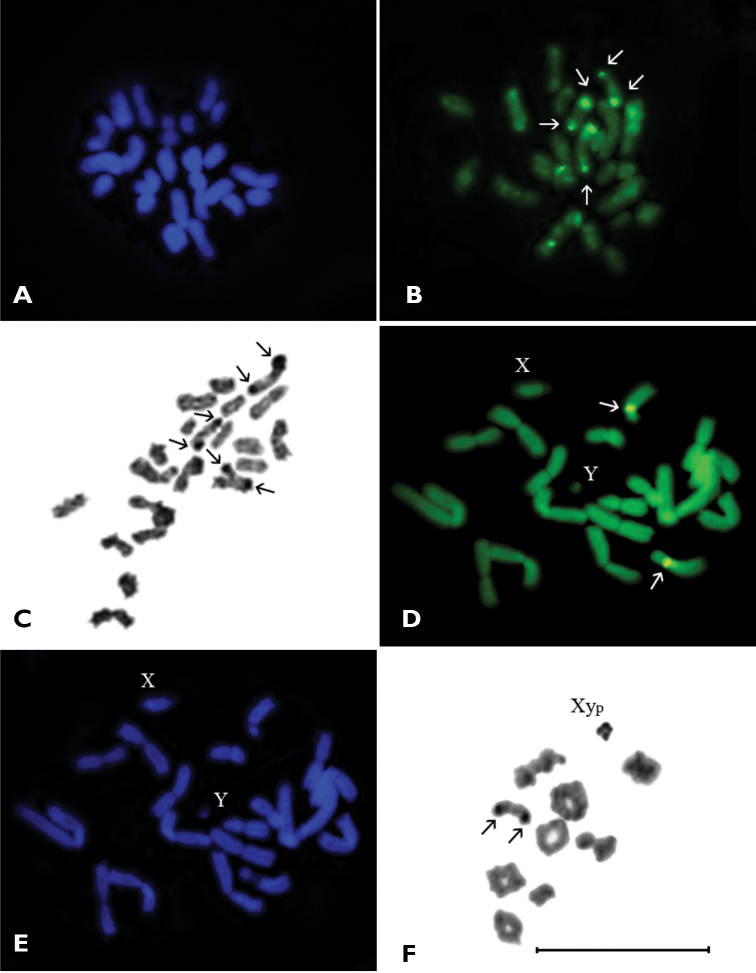
**A–C** *Sphenoptera scovitzii*, n=19-23, metaphase I. **A** DAPI staining; **B** CMA_3_ staining; **C** Ag-banding **D–F** *Dicerca aenea validiuscula*, n=9AA+Xy_p_ **D** mitotic metaphase (CMA_3_ staining) **E** mitotic metaphase (DAPI staining) **F** diakinesis/prometaphase I (Ag-banding). Bar = 10 µm.

Some of chromosomes have a very small amount of constitutive heterochromatin weakly visible in pericentromeric regions and do not form distinct blocks. The DAPI staining of chromosomes did not reveal any positive signal (Fig. 2A), yet fluorescence after CMA_3_ staining was discovered (Fig. 2B). In metaphase I, three or sometimes four rod-shaped bivalents showed distinct CMA_3_ positive signals on telomeric regions of both homologues. These signals are quite stable in the largest and in two of the middle-sized bivalents and correspond to Ag-positive material revealed by the AgNOR-banding technique (Fig. 2C). Weak CMA_3_ positive signals were also at times visible in some other small bivalents.

### Tribe Dicercini

***Dicerca aenea validiuscula***, 2n=20 (18+Xy_p_).

The male mitotic metaphase displayed 20 chromosomes including 9 autosomal pairs and X- and Y- sex chromosomes (Fig. 2D). All autosomes were biarmed: one pair large and 5 pairs of middle-sized metacentrics, 2 pairs of middle-sized submetacentrics and 1 pair of middle-sized subtelocentric. The X-chromosome was middle-sized and acrocentric, Y-chromosome was dot-like with unclear morphology. In mitotic metaphase CMA_3_ positive signals were found in the pericentromeric region of long arm of middle-sized homologous pair of subtelocentric autosomes (Fig. 2D). The DAPI staining did not reveal any positive signal (Fig. 2E).

**Figure 3A–F.**
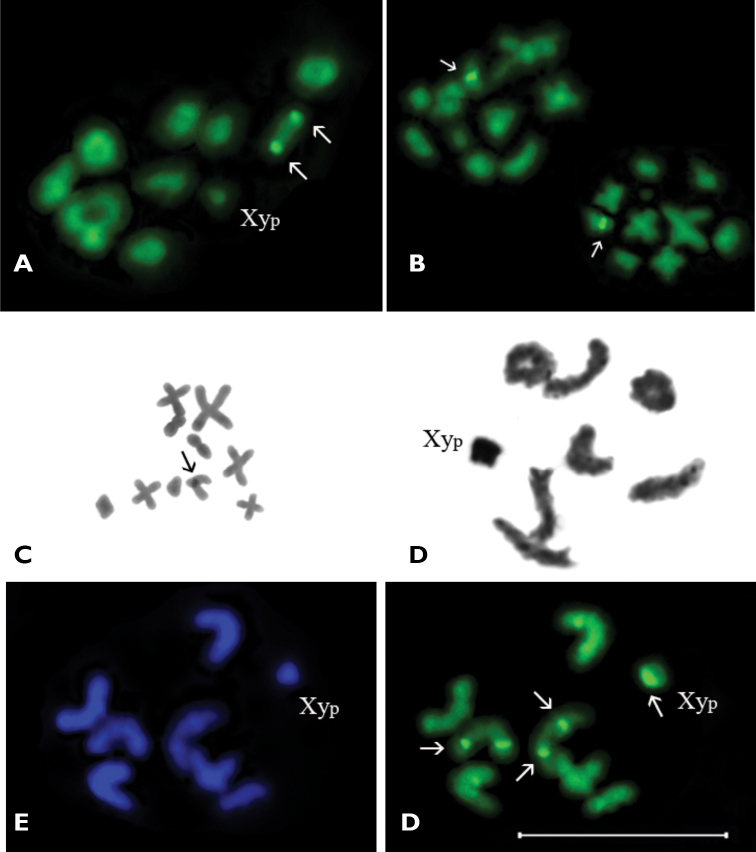
**A–C** *Dicerca aenea validiuscula*,**A** diakinesis/prometaphase I (CMA_3_ staining) **B** metaphase II (CMA_3_ staining) **C** metaphase II (C-banding) **D–F** *Sphaerobothris aghababiani*, n=7AA+Xy_p_ **D** diakinesis/prometaphase I (Ag-banding) **E** metaphase I (DAPI staining) **F** metaphase I (CMA_3_ staining). Bar = 10 µm.

In diakinesis/prometaphase I nine autosomal bivalents and heteromorphic sex-bivalent most probably of “parachute” Xy_p_ type were observed (Figs 2F, 3A). The autosomal bivalents formed a series of gradually decreasing sizes. There were 5–6 ring-shaped autosomal bivalents with two chiasmata, 1–2 rod-shaped bivalents possessed most likely one terminal chiasma and 1–2 were cross-shaped with an interstitial chiasma. The heterovalent Xy_p_ was rather small. In the middle-sized rod-shaped bivalent (which, most likely, formed by subtelocentric autosomes) the CMA_3_ positive signals were visible in terminal regions of both homologues (Fig. 3A). At this stage staining by AgNOR-banding revealed Ag-positive signals on the same bivalent, besides the sex bivalent was nearly homogenously argentophilic (Fig. 2F).

Metaphase II showed 10 chromosomes of which 7 chromosomes were meta- and submetacentric, one of chromosome was subtelocentric (which showed CMA_3_ positive signal on pericentromeric region), whereas the morphology of other chromosomes was vague (Figs 3B, 3C). The C-banding revealed weakly visible constitutive heterochromatin localized in the pericentromeric region of the same subtelocentric chromosome (Fig. 3C).

### Subfamily Buprestinae. Tribe Chrysobothrini

***Sphaerobothris aghababiani***, 2n=16 (14+Xy_p_).

In diakinesis/prometaphase I seven autosomal bivalents and a sex chromosome heterovalent of the Xy_p_ type were observed (Fig. 3D). The bivalents gradually decreased in size. The Xy_p_ sex heterovalent was smallest element in the set. The majority of autosomal bivalents were rod-shaped, but in some cells one or two ring-shaped autosomal bivalents were observed.

The C-banding revealed a small amount of constitutive heterochromatin most probably in the pericentromeric regions and do not form distinct blocks. The DAPI staining did not reveal any positive signals (Fig. 3E). In metaphase I bright and distinct CMA_3_ positive signal was visible on “parachute” sex heterovalent, probably connected with the Y-chromosome (Fig. 3F). In addition, weak CMA_3_ positive signals were observed, as a rule they were situated in telomeric or near telomeric regions of some of rod-shaped autosomal bivalents. Ag-banding revealed the bright argentophilic material connected with whole “parachute” type sex heterovalent (Fig. 3D). Small and weak argentophilic blocks were visible in telomeric or near telomeric regions of some autosomal bivalents.

## Discussion

In total, the karyotypes of 92 species of jewel-beetles belonging to 23 genera, 14 tribes of 5 subfamilies are presently described. Generalization of data including new ones shows that the modal diploid chromosome number in Buprestidae is 2n=20 (9AA + X- and Y- sex chromosome heterovalent in males) so far found in 18 species belonging to 8 genera, 6 tribes, 4 subfamilies. The Xy_p_ type of sex chromosome heterovalent is modal and occurs in 66 species, 16 genera, 10 tribes, 4 subfamilies. The most common karyotype 2n=20 (19+Xy_p_) can be considered as modal. The new data confirm modality of this karyotype within the family. This karyotype occurs in a large number of beetles from different families (Smith and Virkki 1978; James and Angus 2007; Schneider et al. 2007; Cabral-de-Mello et al. 2008; et al.).

Application of C-banding technique showed that the studied species of jewel-beetles are characterized by a small amount of heterochromatin localized, most probably, in pericentromeric regions of chromosomes, as in most studied species of the order Coleoptera (Smith and Virkki 1978; Rożek et al. 2004; Lachowska et al. 2009; Arcanjo et al. 2009; et al.). On the other hand, in *Euchroma gigantea* large C-blocks were observed in multiple sex chromosomes (X_1_X_2_X_3_-Y_1_Y_2_Y_3_) (Moura et al. 2008). The presence of large C-blocks in chromosomes is uncommon in Coleoptera as a whole and has been observed in a few species only (Juan et al. 1991; Rożek 1992; Rożek and Rudek 1992; Plohl et al. 1993; Rożek and Lachowska 2001, 2003; Rożek et al. 2004; Bione et al. 2005a; Cabral-de-Mello et al. 2010).

Until now, the karyotypes of only seven species of jewel-beetles studied by using AgNOR-staining technique have been published (Karagyan 2001; Moura et al. 2008). In the present paper Ag-banding was applied for study of karyotypes of another three species.

Even this restricted material showed noticeable variability of distribution of argentophilic material (probably NOR) in the karyotypes of jewel-beetles. The argentophilic material is located on:

1) the autosomes: *Sphenoptera scovitzii* (2n=38–46), *Sphenoptera mesopotamica* Marseul, 1865 (2n=24, Xy_p_).

2) both on the sex chromosomes and on the autosomes: *Acmaeoderella villosula* Steven, 1830 (described as *Acmaeoderella boryi* Brullé, 1832 in Karagyan 2001) (2n=18, Xy_r_), *Acmaeodera pilosellae persica* (2n=20, neo-XY), *Sphaerobothris aghababiani* (2n=16, Xy_p_), *Dicerca aenea validiuscula* (2n=20, Xy_p_).

3) sex chromosomes only, situating either on one of the sex chromosomes or localized between sex chromosomes of bivalent: *Acmaeoderella flavofasciata* (Piller & Mitterpacher, 1783) (2n=18, Xy_r_), *Acmaeoderella gibbulosa* Ménétriés, 1832 (2n=18, Xy_r_), *Acmaeoderella vetusta* (Ménétriés, 1832) (2n=18, Xy_r_). In *Euchroma gigantea* (2n=32, 36, X_1_X_2_X_3_Y_1_Y_2_Y_3_ – Moura et al. 2008) argentophilic material labeled the multiple sex chromosomes chain.

As commonly understood, in Coleoptera NORs may be located in some autosomal pair and/or sex chromosomes (Almeida et al. 2000; Moura et al. 2003; Bione et al. 2005b; Holecová et al. 2008). The most common pattern in Coleoptera is the location of the nucleolus organizer region in one autosomal pair (Virkki 1983; Virkki et al. 1984; Vitturi et al. 1999; Colomba et al. 2000; Moura et al. 2003; Almeida et al. 2006; Schneider et al. 2007; et al.). On the other hand, the argentophilous body has been repeatedly observed between the sex-chromosomes of Xy_p_ type (Postiglioni and Brum-Zorrilla 1988; Virkki et al. 1990, 1991; Maffei et al. 2001; Moura et al. 2003; et al.). Among Buprestidae, the whole Xy_p_ sex bivalent is brightly argentophilic and probably bears NOR only in *Sphaerobothris aghababiani* and *Dicerca aenea validiuscula*. However, the lack of relationship between nucleolus and sex chromosome system of Xy_p_ has also been demonstrated for some Coleoptera including one species of jewel-beetles, *Sphenoptera mesopotamica* (Karagyan 2001). According to some authors, the “parachute” can be strongly marked by the silver nitrate during different phases of meiosis independent of whether or not the NORs are located in Xy_p_ bivalent (Virkki et al. 1991; Moura et al. 2003; Bione et al. 2005b; Mendes-Neto et al. 2010). This phenomenon is probably related to the presence of argentophilic substance (proteins) that theoretically facilitates the configuration and segregation of the sex chromosomes of the Xy_p_ system (Virkki et al. 1990, 1991; Juan et al. 1993; Petitpierre 1996; Moura et al. 2003; Bione et al. 2005a, b; Schneider et al. 2007).

Chromosome staining by DNA base specific fluorochromes has been used in cytogenetic studies of Coleoptera (Juan and Petitpierre 1989; Vitturi et al. 1999; Colomba et al. 2000; Moura et al. 2003; Schneider et al. 2007; Lachowska 2008; Mendes-Neto et al. 2010; et al) but has never before been applied to Buprestidae.

The correlation between NORs and CMA_3_ bands is quite common in some insects, including beetles (Camacho et al. 1991; Vitturi et al. 1999; Colomba et al. 2000; Maffei et al. 2001; Kuznetsova et al. 2001; Araújo et al. 2002; Brito et al. 2003; Nechayeva et al. 2004). Silver staining mainly reveals transcriptionally active NORs (Sumner 1990), as opposed to fluorochrome CMA_3_ staining which labels NORs independently of their activity (Schmid and Guttenbach 1988). In this study, the fluorescent signals after CMA_3_ staining were positive in all four species of jewel-beetles. While in *Sphenoptera scovitzii* and *Sphaerobothris aghababiani* they were nearly fully correlated with argentophilic material observed on silver dyed chromosomes, in *Acmaeodera pilosellae persica* CMA_3_ signals correlated with argentophilic blocks on 2–3 autosomal bivalents but not with Y-chromosome which was dyed argentophilic as well. Meanwhile in *Dicerca aenea validiuscula* CMA_3_ signal was correlated with one of autosomal pairs when argentophilic material was revealed in the same autosomal pair as well as on sex bivalent.

Fluorochrome DAPIstaining in all studied species of jewel-beetles did not reveal any particular bright regions on chromosomes which allows for preliminary suggestion that CH have no distinct AT-rich DNA clusters. In general, there is variable distribution of AT- or GC-rich clusters of CH among beetles studied by fluorochromes staining. For instance, positive CMA_3_ and negative DAPI signals were found in some Elateridae (Schneider et al. 2006, 2007), and most of studied Scarabaeoidea (Vitturi et al. 1999; Moura et al. 2003; Bione et al. 2005b; Cabral-de-Mello et al. 2010). Positive DAPI signals were found only in some other Scarabaeoidea (Moura et al. *l. c*.) and in majority of the Curculionidae studied (Lachowska 2008; Lachowska et al. 2008). Rarely both DAPI and CMA_3_ positive signals were revealed in Scarabaeidae (Bione et al. 2005b), in a few Curculionidae (Lachowska 2008) and Chrysomelidae (Almeida et al. 2006).

In conclusion, data of the present study offer important insights into the karyotypes characteristics of jewel-beetles which may be useful in elucidation of relationships both among the species of the family itself as well as between jewel-beetles and the representatives of other coleopteran families.
